# Editorial: Secretomics: More Secrets to Unravel on Plant-Fungus Interactions

**DOI:** 10.3389/fpls.2020.601021

**Published:** 2020-10-21

**Authors:** Jan Schirawski, Maryam Rafiqi, Dominique Job, Martijn Rep, Delphine Vincent

**Affiliations:** ^1^Genetics, Matthias-Schleiden-Institute, Friedrich-Schiller-University Jena, Jena, Germany; ^2^AgroBioSciences Program, Mohammed VI Polytechnic University (UM6P), Ben Guerir, Morocco; ^3^CNRS/Université Claude Bernard Lyon 1/Institut National des Sciences Appliquées/Bayer CropScience Joint Laboratory (UMR 5240), Bayer CropScience, Lyon, France; ^4^Molecular Plant Pathology, Swammerdam Institute for Life Sciences, University of Amsterdam, Amsterdam, Netherlands; ^5^Agriculture Victoria Research, AgriBio, Center for AgriBioscience, Bundoora, VIC, Australia

**Keywords:** plant-fungus-interactions, extracellular vesicles (EVs), effectors, secretion, effector function

Plants interact with microbes (both beneficial and pathogenic) in many ways. As we begin to gain an understanding of the many molecular mechanisms that shape these interactions, it becomes clear that a variety of molecules serve as key interchange in signaling between microbes and plants.

## Extracellular Vesicles in Plant-Fungal Communication

As we learn in an instructive review on the topic by Vincent et al., molecules are exchanged in a bidirectional flow between plants and microbes and are very diverse in nature, ranging from complex polymers (e.g., proteins, nucleic acids) to small compounds (e.g., ATP, phytohormones, phytochemicals, secondary metabolites). In many cases, extracellular vesicles (EVs) serve as a means of molecule transport. They constitute small, membrane-enclosed structures that are released from a cell into the surrounding environment. By secretion and uptake of extracellular vesicles, various cargoes are transported from the cytoplasm of a donor cell to that of an acceptor cell. This way of communication does not only constitute a protein secretion pathway that is alternative to the classical route of protein delivery via the endoplasmic reticulum, but also allows the transport of many additional molecules other than proteins. Thus, this review makes a strong case of considering more than just proteins when seeking to understand plant-microbe communication. This is of invaluable agricultural importance because of the phytopharmaceutical potential of plant and/or artificial EVs, since they can be used to deliver compounds promoting crop growth and resilience to pathogen infection, as is currently investigated in medicine.

First observed in plant cells, EVs can also be delivered by fungal cells, as shown in a research article by Bleackley et al.. EVs isolated from cultures of the major cotton pathogen *Fusarium oxysporum* f. sp. *vasinfectum* contain various proteins in addition to a naphthoquinone pigment. Numerous proteins were found to be delivered this way, including polyketide synthases, proteases and many proteins with an as yet unknown function. Isolated fungal EVs were found to be phytotoxic when applied to plant leaves, suggesting that EVs may be used by this hemibiotrophic vascular wilt pathogen to deliver proteins or secondary metabolites with a role in the infection process.

EVs also play a role in the interaction of *Botrytis cinerea* with its host plants. To understand factors contributing to the virulence of this necrotrophic ascomycete, de Vallée et al. generated and compared a set of non-pathogenic *B. cinerea* mutants. Interestingly, investigation of the secretome of four non-pathogenic mutants revealed a common profile of down-regulated lytic enzymes. For eight additional non-pathogenic mutants, deficiencies in the secretion of proteases and hemicellulases were observed. This strongly implicates these secreted enzymes and the secretion process itself in the virulence of *B. cinerea*.

## Searching for Effector Effects

Four research papers in this Research Topic strive to identify the effects of fungal effector proteins in diverse plant-fungal pathosystems. Dutra et al. searched for effectors of the biotrophic head smut pathogen of maize, *Sporisorium reilianum* f. sp. *zeae*, with a putative effect on suppression of induced plant cell death. The authors screened a set of 62 predicted effector proteins by transient expression in *Nicotiana benthamiana*, both alone and in combination with the cell death-inducing protein INV1 of *Phytophthora infestans*. This has led to the identification of a fungal effector able to efficiently suppress INV1-induced cell death.

To identify effectors with a putative role in suppressing cell death or plant immunity, Qi et al. used a heterologous expression assay in *N. benthamiana* to screen 31 putative effectors of the biotrophic rust fungus of common bean, *Uromyces appendiculatus*. The putative effectors were screened for their subcellular localization and for their ability to suppress cell death in yeast, as well as for their ability to suppress PAMP- or effector-triggered immunity in *N. benthamiana*. The authors identified several effectors with a putative role in suppressing plant defenses that, interestingly, were not necessarily up-regulated upon *U. appendiculatus* colonization of its host plant *Phaseolus vulgaris*. This may indicate that these effectors are primarily used at fungal life stages different than those investigated, or alternatively, that strong upregulation of their expression during in-planta growth may not be necessary for their function. The latter, in turn, would indicate that up-regulation during plant colonization may not be as important a criterion for identification of effectors as previously thought.

Looking at the interaction capacity of effectors of the biotrophic common maize smut fungus *Ustilago maydis*, Alcantâra et al. used a systematic yeast two-hybrid interaction screen to find that more than a third of the 300 tested effectors interacted either with themselves or with other effectors, which suggests that many effectors may function in complexes. This is an interesting finding that needs to be considered when trying to elucidate effector functions in this pathosystem and other ones.

Lastly, to gain insight into the evolution of effector gene repertoires in the context of host specialization, Kim et al. analyzed effector distribution, sequence variation and genomic context of five pathotypes of *Magnaporthe oryzae*, three isolates of *Magnaporthe grisea*, and eight additional strains representing different species of the Magnaporthaceae. The authors found indications of a faster evolution of effector candidates than of single copy orthologs, as well as a large variation in effector gene repertoire between these species, likely caused by adaptation to their different hosts.

## Secretomics for Future

Together, these articles indicate that the secretion of effectors and other molecules by the classical secretion apparatus as well as through extracellular vesicles is crucial for the communication between microbes and plants. This calls for more comprehensive studies addressing the large topic of secretion on both sides of the plant-microbe interface. The era of secretomics has begun. And, as is evident in its name, secretomics still contains the one or the other secret that needs to be unraveled.

## Author Contributions

JS wrote the first draft of the editorial. All authors edited the manuscript and approved the final version.

## Conflict of Interest

The authors declare that the editorial was written in the absence of any commercial or financial relationships that could be construed as a potential conflict of interest.

